# Developing and testing a web‐based intervention to encourage early help‐seeking in people with symptoms associated with lung cancer

**DOI:** 10.1111/bjhp.12325

**Published:** 2018-07-12

**Authors:** Julia Mueller, Alan Davies, Caroline Jay, Simon Harper, Fiona Blackhall, Yvonne Summers, Amelie Harle, Chris Todd

**Affiliations:** ^1^ School of Health Sciences University of Manchester UK; ^2^ Manchester Academic Health Science Centre UK; ^3^ School of Computer Science University of Manchester UK; ^4^ Department of Medical Oncology The Christie NHS Foundation Trust Manchester UK; ^5^ Division of Molecular and Clinical Cancer Sciences University of Manchester UK; ^6^ Department of Medical Oncology Poole Hospital NHS Foundation Trust Poole UK; ^7^ Manchester University Foundation NHS Trust UK

**Keywords:** behaviour change techniques, digital health, health information seeking, help‐seeking behaviour, lung cancer, online health information, tailoring, theory of planned behaviour, web‐based intervention

## Abstract

**Objectives:**

To detail the development method used to produce an online, tailored, theory‐based, user‐centred intervention to encourage help‐seeking for potential lung cancer symptoms.

**Design:**

Intervention development was structured around the person‐based approach. The feasibility study involved a randomized controlled trial design.

**Methods:**

Intervention development drew on qualitative inquiries, the Theory of Planned Behaviour (TPB), and identifying concrete mechanisms of change to implement in the intervention (Behaviour Change Techniques). The final intervention involved two key features: (1) tailoring and (2) ‘TPB components’ to target beliefs about help‐seeking. In an online feasibility study, we recruited people reporting potential lung cancer symptoms using mailing lists, social media, websites, and Google AdWords. Participants were randomized to the intervention, a tailored comparison group (CG) without TPB‐components, an untailored CG with TPB components, or a CG with neither. Following treatment, participants clicked a button to indicate whether they wished to make an appointment and completed a TPB questionnaire.

**Results:**

A total of 130 participants reporting relevant symptoms were recruited (24% of website visitors). Participants in the intervention group reported higher intention to seek help than those who received tailored information without TPB components (*p *=* *.03). User comments indicate more support is needed for people who sought help for symptoms, but felt dismissed.

**Conclusions:**

The potential for differential dropout in online randomized trials requires careful consideration. Future help‐seeking interventions should provide support for those who have previously felt dismissed by health professionals. The feasibility study provides some evidence that our ‘TPB components’ were effective, but validation in a powered trial is necessary.

**Statement of contribution**

***What is already known on this subject?***

- People with lung cancer often delay presenting symptoms to health services.
- Some patients (or their family/friends) look up symptoms online before their diagnosis, to decide whether they should see a doctor.
- Interventions are needed to ensure people can find useful information online that will encourage them to seek help for relevant symptoms.

***What does this study add?***

- Theory‐mapping and user involvement facilitated systematic intervention development.
- Lung cancer help‐seeking interventions should address salient beliefs and personal relevance.
- The potential for differential dropout in online randomized trials requires careful consideration.

## Background

More people die of lung cancer each year than any other cancer types (Fitzmaurice *et al*., 2015). Lung cancer survival rates in the United Kingdom are lower than in other European countries that have comparable per capita total national expenditure for health (De Angelis *et al*., 2014; Holmberg *et al*., 2010). Research indicates these low survival rates are attributable to delays to diagnosis (Holmberg *et al*., 2010). Delays to diagnosis can lead to advanced stage of disease when treatment is commenced (Radzikowska, Roszkowski‐Sliz, & Glaz, 2012), which decreases treatability and chance of survival (Zee, Eisen, & Carney, 2008). It is estimated that every year up to 1,300 lung cancer deaths could be avoided if the United Kingdom matched the highest survival rates in Europe (Richards, 2009).

The time to diagnosis depends on several factors including patient, health care provider, and system factors as well as disease factors (Walter, Webster, Scott, & Emery, 2012). The Pathways to Treatment model theorizes the time between first symptom occurrence and diagnosis into three intervals, involving symptom appraisal, help‐seeking, and diagnostic procedures (Scott, Walter, Webster, Sutton, & Emery, 2013). Early diagnosis in lung cancer relies mainly on recognition and presentation of warning signs by patients, but awareness of lung cancer symptoms among the public in the United Kingdom appears to be low (Simon *et al*., 2012).

Research has indicated that patients in the United Kingdom experience symptoms for several months before presenting to health services (Corner, Hopkinson, Fitzsimmons, Barclay, & Muers, 2005; Corner, Hopkinson, & Roffe, 2006; Tod, Craven, & Allmark, 2008). Reasons for delayed presentation include lack of awareness of symptoms, attribution of symptoms to minor health conditions (e.g., a common cold, a smoker's cough) or ageing, and sometimes fear of finding a serious cause and fatalistic beliefs about lung cancer (Brindle, Pope, Corner, Leydon, & Banerjee, 2013; Corner *et al*., 2005, 2006; Lyratzopoulos, Liu, Abel, Wardle, & Keating, 2015; McCutchan, Wood, Edwards, Richards, & Brain, 2015; McLachlan *et al*., 2015; Shim, Brindle, Simon, & George, 2014; Smith, Pope, & Botha, 2005; Tod & Joanne, 2010; Walton *et al*., 2013).

Several public health campaigns have been launched in recent years in the United Kingdom to promote help‐seeking for lung cancer symptoms, for example ‘Be Clear on Cancer’ and ‘Detect Cancer Early’ (Gordon, Magee, Jones, Phillipson, & Barrie, 2012; Ironmonger *et al*., 2014). These campaigns usually disseminate brief, simple messages mostly addressing the cough symptom. Although they tend to result in increased knowledge levels, the symptom appraisal/help‐seeking interval often remains the same (Tustin, 2012). People with relevant symptoms who are aware of the campaigns sometimes describe not perceiving the information as relevant to themselves (Caswell *et al*., 2017; McLachlan *et al*., 2015). Furthermore, due to the campaigns’ strong focus on the cough symptom, people often remain unaware of other warning signs such as backache, weight loss, or fatigue (Birt *et al*., 2014; Caswell *et al*., 2017; McLachlan *et al*., 2015). Walter *et al*. (2015) recommend that initiatives for improving lung cancer awareness should focus on multiple symptoms rather than single symptoms, as single symptoms rarely predict lung cancer reliably.

Thus, other intervention forms are needed which are perceived as personally relevant, and which provide detailed information on the varied symptom profile of lung cancer. Considering the rapidly increasing volume of health information on the web and the increasing tendency of individuals to seek health information online, the web could potentially be a key factor influencing awareness and health decisions. Individuals often research their symptoms prior to presentation to health services, and this can impact on decisions to seek help (Mueller *et al*., 2017). Moreover, recent research shows some people with lung cancer report researching their symptoms online prior to being diagnosed, and this seems to affect decisions of whether to present to health services and whether to request further diagnostic tests (Mueller, Jay, Harper, & Todd, 2017). Thus, targeting online health information could influence lung cancer symptom awareness and delays to diagnosis, ultimately improving chances of survival.

At present, web pages about lung cancer symptoms typically present a list of possible symptoms and a brief description of risk factors (e.g., NHS Choices, n.d). However, to our knowledge, no study has to date assessed whether this is the ideal form of presenting lung cancer information in order to ensure prompt help‐seeking for warning signs.

To address the question of whether a new way of presenting online health information about lung cancer could increase help‐seeking, we developed an online intervention designed to encourage early help‐seeking based on psychological theory and designed a study to evaluate its effectiveness. This study details the methods used to develop our intervention, highlighting how we translated insights from theory into practice, and presents results from a feasibility study. This detailed description of the intervention development process is important because information on the content and design of interventions for health behaviours is often lacking (Glasziou, Meats, Heneghan, & Shepperd, 2008; McCleary, Duncan, Stewart, & Francis, 2013), rendering findings difficult to reproduce or use for future research and practice (Wood *et al*., 2015). Where details regarding intervention development are published, these provide extremely useful insights (Smith *et al*., 2012; Smits *et al*., 2016). In particular, the evidence base for online health interventions such as mobile apps and health websites is often limited (DiFilippo, Huang, Andrade, & Chapman‐Novakofski, 2015; Kampmeijer, Pavlova, Tambor, Golinowska, & Groot, 2016; McKay *et al*., 2018; Rogers, Lemmen, Kramer, Mann, & Chopra, 2017; Zhang, Sun, & Xie, 2015; Zhao, Freeman, & Li, 2016). More thorough documentation and evaluation of methods are required to promote more evidence‐based approaches in this rapidly growing field.

### Intervention design

#### Overview

Intervention development was informed by the person‐based approach (Yardley, Morrison, Bradbury, & Muller, 2015). The first step entailed drawing on previous relevant research and patient and public involvement (PPI) to understand the context and the main barriers to help‐seeking in lung cancer. This also led to the formulation of a set of guiding principles which were consulted throughout the development of the intervention, as is recommended in the person‐based approach (Yardley *et al*., 2015). Step two focused on targeting relevant beliefs by drawing on psychological theory. The third step involved developing and operationalizing a tailoring strategy to tailor our intervention to individual users. Finally, in the fourth step, we evaluated the intervention in a Think Aloud usability study.

### Step 1: Preliminary work and preparations

As suggested by Yardley *et al*. (2015), we used a combination of qualitative research and a set of guiding principles to develop an acceptable and persuasive intervention, paying attention to the particular context of the target population.

#### Drawing on interviews and previous relevant research

To gain initial insights into the needs of people with lung cancer who use the web to appraise symptoms prior to diagnosis, we conducted interviews with patients with lung cancer and their family/friends (Mueller, Jay, Harper, & Todd, 2017). We found that the web is reportedly used in all intervals leading up to diagnosis in the Pathways to Treatment model (Scott *et al*., 2013). This informed our decision to develop a web‐based intervention to promote earlier help‐seeking. We also drew on previous qualitative research on help‐seeking for lung cancer symptoms (Birt *et al*., 2014; Braybrook, Witty, & Robertson, 2011; Caswell *et al*., 2017; Corner *et al*., 2006; McLachlan *et al*., 2015; Smith *et al*., 2005; Tod *et al*., 2008). Additionally, we conducted a systematic review on web use for symptom appraisal (Mueller *et al*., 2017). In the research team, we discussed findings from these different sources and their implications for the intervention and brainstormed for initial ideas of how these could be addressed. This resulted in an initial set of ideas for intervention components to address user needs and perspectives (Table 1).

**Table 1:** Incorporation of findings from previous studies into the development of the intervention

Findings from previous studies	Implication for the intervention	How could this be addressed in the intervention?
People are aware of previous lung cancer awareness campaigns, but mostly cough (not other symptoms), and not very detailed knowledge (Birt *et al*., 2014; Caswell *et al*., 2017; McLachlan *et al*., 2015)	We need to find a way to present information about other symptoms as well without overwhelming users	Elicit symptoms from individual users first and provide more in‐depth information on these (tailored information)
Some people aware of previous lung cancer awareness campaigns assume the information is not relevant to them (Caswell *et al*., 2017; McLachlan *et al*., 2015)	We need to make sure the information presented is perceived as personally relevant	Present individually tailored information
Family/friends play an important role in help‐seeking in lung cancer and are often the trigger for initial consultation (Braybrook *et al*., 2011; Corner *et al*., 2006; Smith *et al*., 2005; Tod *et al*., 2008)	We need to enhance users’ belief that other significant people (family/friends) want them to seek medical advice	Emphasize message is endorsed by family/friends
People tend to use a ‘process of elimination’ to diagnose symptoms online: comparing symptoms against those listed and discarding conditions for which the match is low (likely to happen with lung cancer as people typically only display 1–3 symptoms) (Mueller, Jay, Harper, Davies, *et al*., 2017)	Rather than present individuals with a list of symptoms, we should present them with specific details on *their* particular symptoms	Personalization of symptom information, presenting detailed information on endorsed symptoms
People use online health information to prepare for consultations (Mueller, Jay, Harper, Davies, *et al*., 2017); this is also documented for patients with lung cancer (Mueller, Jay, Harper, & Todd, 2017)	We need to provide users with information they can easily take to their next consultation	Printable personalized summary of symptoms, risk factors, and recommendations
People with lung cancer use online health information during the diagnostic interval, to support claims to their GP that further investigation of their symptoms is warranted (Mueller, Jay, Harper, & Todd, 2017)	We need to provide users with guidance on when symptoms warrant further investigation and information that can help them communicate with health professionals	Personalized information on NICE guidelines for suspected cancer referral
People tend to trust websites of well‐known organizations (Mueller, Jay, Harper, & Todd, 2017)	We need to enhance trust by showing that our message is endorsed by NHS health professionals We need to emphasize collaboration with organizations people know	Emphasize message is endorsed by health professionals Mark all pages with the University of Manchester and Medical Research Council logo. Emphasize that NHS practitioners reviewed the intervention

#### Patient and public involvement (PPI)

Based on the exploration of previous research (Table 1), we concluded that the intervention should include two factors: (1) tailoring of information in order to enhance perceived personal relevance, mitigate the ‘process of elimination’ (Table 1), and present information on multiple symptoms (not only cough) without overwhelming users; and (2) incorporation of components to address key beliefs that may impact on execution of behaviour.

We discussed the conceptualized ideas and proposed content with members of a PPI group consisting of people who have been affected by cancer (i.e., either they or someone among their friends/family have, or had, cancer). This group meets regularly at a University in Northwest England and provides PPI input for researchers. Before the meeting, the group members were sent a Powerpoint file that contained a rough draft of the intervention and requested to consider the following:

How can the information be personalized so that it addresses individual needs and appears personally relevant?
How can we present information to influence/change peoples’ beliefs?
Is the proposed information clear and easy to understand?
  ○  If not, how could this be clarified?
2 Is the proposed phrasing acceptable?
  ○  If not, how could it be phrased more clearly?
2 How can we make the website appear credible and professional?

Curtis, Lahiri, and Brown (2015) recommend incorporating user suggestions that are (1) in line with the target behaviour, (2) compatible with the theoretical basis of the intervention, (3) compatible with usability recommendations, and (4) easy to implement online. We used these criteria to decide whether to ‘accept’ or ‘reject’ user preferences during usability and PPI work. The main points derived from the PPI work are shown in Box 1.

Box 1 **Suggestions made by the Patient and Public Involvement group/thoughts voiced by the group regarding our initial ideas**

1

- *Professional credibility:* Confidence in the information provided is very important. A specific page should be dedicated to providing information about the research team, where the medical information and recommendations were derived from, who reviewed the website etc.
- *Endorsement by health professionals:* The group suggested that some form of source credibility should be added to enhance impact on peoples’ beliefs, that is, endorsement by health professionals or other relevant people. The group therefore suggested including quotes by health professionals, accompanied by names and affiliations, to emphasize key messages.
- *Avoiding the term cancer:* The group pointed out that the term ‘lung cancer’ could cause anxiety and distress. They suggested putting stronger focus on the symptoms and when to seek help and avoiding the term cancer wherever possible.
- *Avoiding self‐diagnosis:* The group raised the possibility that users might use the intervention as a self‐diagnosis tool. They suggested emphasizing that the website is designed to provide advice on when to seek help, rather than diagnose a disease.
- *Medical disclaimer:* The group recommended emphasizing to users that the website should not be used to replace advice from a medical professional.
- *Clear terminology:* There was some confusion regarding terminology used to refer to health professionals. The group recommended using the term ‘doctor’ consistently, possibly with General Practitioner/GP in brackets.

#### Guiding principles

According to the person‐based approach (Yardley *et al*., 2015), it is useful to produce a set of guiding principles which can be consulted throughout planning and development of an intervention. Our guiding principles were informed by our interview study on pre‐diagnosis web use among patients with lung cancer (Mueller, Jay, Harper, & Todd, 2017) as well as previous research on help‐seeking in lung cancer (Birt *et al*., 2014; Corner *et al*., 2005, 2006; McLachlan *et al*., 2015; Tod & Joanne, 2010; Tod *et al*., 2008) (Box 2).

Box 2 **Guiding principles**

1 1. Intervention objectives

- To provide information to people with symptoms potentially related to lung cancer and encourage medical help‐seeking where appropriate.

2. Relevant aspects of users in the context

People with lung cancer are often:
  ○  Older
  ○  Less technically literate
  ○  Of lower socioeconomic/educational level
2 Those looking up symptoms may be family members/friends rather than afflicted individuals themselves

3. Key behavioural issues, needs, or challenges that the intervention needs to address

- Barriers to help‐seeking identified in the literature:
  ○  Symptoms are experienced as too mild to warrant help‐seeking
  ○  Symptoms are perceived as related to existing conditions
  ○  People think symptoms of lung cancer would be more severe
  ○  Poor knowledge of risk factors
  ○  Fear and fatalistic beliefs about lung cancer and treatability
  ○  Fear of wasting the doctor's time/being seen as a time‐waster
  ○  Fear of blame and stigma (due to smoking)
  ○  Culture: Great value placed on stoicism, media advice not to present to primary care unless severe
  ○  Dislike of attending doctor's surgery
  ○  Difficulties accessing health care (e.g. competing responsibilities and limited availability of appointments)
  ○  Information (e.g., from media campaigns) perceived as irrelevant to the individuals’ situation

4. Intervention design features that can address the barriers and achieve the aim

Tailoring
  ○  Will ensure users are informed about their specific symptoms and risk factors and when help‐seeking is warranted
  ○  Will make the information appear more personally relevant
2 Theory‐based components
  ○  Will address beliefs such as fear, fatalistic beliefs, fear of wasting the doctors’ time, and fear of social stigma
2 Provide information on when a referral for a chest x‐ray is warranted
  ○  To help individuals communicate with their doctors during the diagnostic process
2 The intervention should be kept as close as possible to the usual experience of using the web to appraise symptoms (as we are targeting people engaging in this activity), that is, brief, one‐off visit to the website, no multiple visits required

Once we had created a draft of the intervention design with the PPI group and established guiding principles, we proceeded to develop the intervention website. In the following section, we describe how we implemented theory and how information was tailored.

### Step 2: Incorporating theory and targeting relevant beliefs

To target beliefs about help‐seeking, we drew on the Theory of Planned Behaviour (TPB) (Ajzen, 1985). Our reasons for focusing on TPB include the following:

a TPB has been successfully used to predict help‐seeking for cancer symptoms (Hunter, Grunfeld, & Ramirez, 2003),
b A meta‐analysis of Internet‐based behaviour change interventions found larger effects for TPB than other theoretical approaches (Webb, Joseph, Yardley, & Michie, 2010),
c TPB has been successfully used to inform the development of a previous (non‐Web‐based) intervention to target help‐seeking in lung cancer (S. M. Smith *et al*., 2012),
d TPB takes an individual's perception of their social environment, consequences of the behaviour, and control over the behaviour into account, and previous literature shows these are key factors influencing lung cancer patients’ decisions to seek medical help (Birt *et al*., 2014; Corner *et al*., 2005, 2006; McLachlan *et al*., 2015; Tod & Joanne, 2010; Tod *et al*., 2008).

The TPB indicates that behaviour is a function of intention to perform the behaviour, which is influenced by beliefs: *behavioural beliefs* about the behaviour and its outcomes, *normative beliefs* about social expectations and motivation to comply with these expectations, and *control beliefs* about the extent of control one has over executing the behaviour. These beliefs in turn influence attitudes towards the behaviour, perceived social norms, and perceived behavioural control.

To target TPB constructs, we proceeded in three stages, described below.

#### (i)Identifying salient beliefs and linking these to TPB constructs

The TPB postulates that *salient* beliefs, that is those that are relevant and accessible in the specific context, will affect intention to perform a behaviour (Ajzen, 1991). We used findings from previous studies on help‐seeking behaviour among people with lung cancer (Birt *et al*., 2014; Corner *et al*., 2005, 2006; McLachlan *et al*., 2015; Tod & Joanne, 2010; Tod *et al*., 2008) to develop a list of beliefs that appear to play a role in help‐seeking for lung cancer symptoms. Next, these beliefs were mapped onto the three types of beliefs identified in TPB (behavioural, normative, and control beliefs) using a consensus‐based approach in the research team (Table 2).

**Table 2:** Salient beliefs about help‐seeking identified from the literature, mapped onto TPB constructs

Beliefs about outcomes	Normative beliefs	Control beliefs
If medical advice is sought, no serious cause will be found. (Birt *et al*., 2014; Corner *et al*., 2005, 2006; McLachlan *et al*., 2015; Tod *et al*., 2008)	Worry about being seen as a time waster by doctors. (Tod & Joanne, 2010)	Perceived difficulties due to limited access to health care/availability of appointment (Birt *et al*., 2014)
Fear and fatalistic beliefs about lung cancer and treatability (seeking medical advice might be pointless if it is lung cancer, because lung cancer cannot be treated) (Tod & Joanne, 2010; Tod *et al*., 2008)	Culture: Great value placed on stoicism, media advice not to present to primary care unless severe (Tod *et al*., 2008; (Tod & Joanne, 2010)	
Worry about wasting the doctors’ time (Tod *et al*., 2008)	Fear of blame and stigma (due to smoking) (Corner *et al*., 2005, 2006; Tod & Joanne, 2010; Tod *et al*., 2008)	

#### (ii)Identifying behaviour change techniques (BCTs) to target TPB constructs

The next step was to consider which techniques to use in order to change these beliefs.

Michie, Johnston, Francis, Hardeman, and Eccles (2008) have developed a comprehensive matrix which maps BCTs, defined as observable mechanisms of change used in behaviour change interventions, onto theoretical construct domains found in a wide array of psychological behaviour change theories. We used this matrix to identify which BCTs to use in our intervention. First, we identified which of the construct domains in Michie *et al*.'s matrix most closely matched the TPB constructs. Two independent researchers undertook this matching exercise, achieving 100% agreement: ‘Beliefs about consequences’ were matched with ‘behavioural beliefs’, ‘Social influences’ were matched with ‘normative beliefs’, and ‘Beliefs about capabilities’ were matched with ‘control beliefs’. We then collated a list of all BCTs considered to be effective in changing these constructs according to Michie *et al*.'s matrix (2008).

We assessed these BCTs for their suitability for the intervention based on the following criteria:

a) Amenability to mode of delivery (web‐based, one‐off short‐term interaction of about 20 min; users will not return to the website at later time).
b) Relevance and suitability for the targeted behaviour: Making an appointment with the GP to have the symptoms checked.
c) Relevance and suitability to target the beliefs identified in Table 2.

The assessment is shown in Appendix A. This led to the identification of the BCTs shown in Table 3.

**Table 3:** Behaviour change techniques (BCTs) identified as suitable and likely to be effective in the TPB‐based intervention. BCTs are numbered to facilitate reference to them in the following text

Construct domain	Maps on to TPB construct	BCT
Beliefs about consequences	Behavioural beliefs	Information regarding behaviour and outcome;
		Persuasive communication
Social influences	Normative beliefs	Social processes of encouragement, pressure, and support
Beliefs about capabilities	Control beliefs	Increasing skills: problem‐solving, decision‐making, and goal‐setting

#### (iii)Operationalizing behaviour change techniques

The BCTs identified above then needed to be operationalized. As Box 1 shows, the PPI group suggested we use quotes to substantiate the messages; thus, we decided to use this medium to convey information to target beliefs. We show examples below of how TPB constructs were targeted using three different ‘TPB components’. Intervention screenshots are shown in Appendix B.

#### Behavioural beliefs

**Figure 1:**
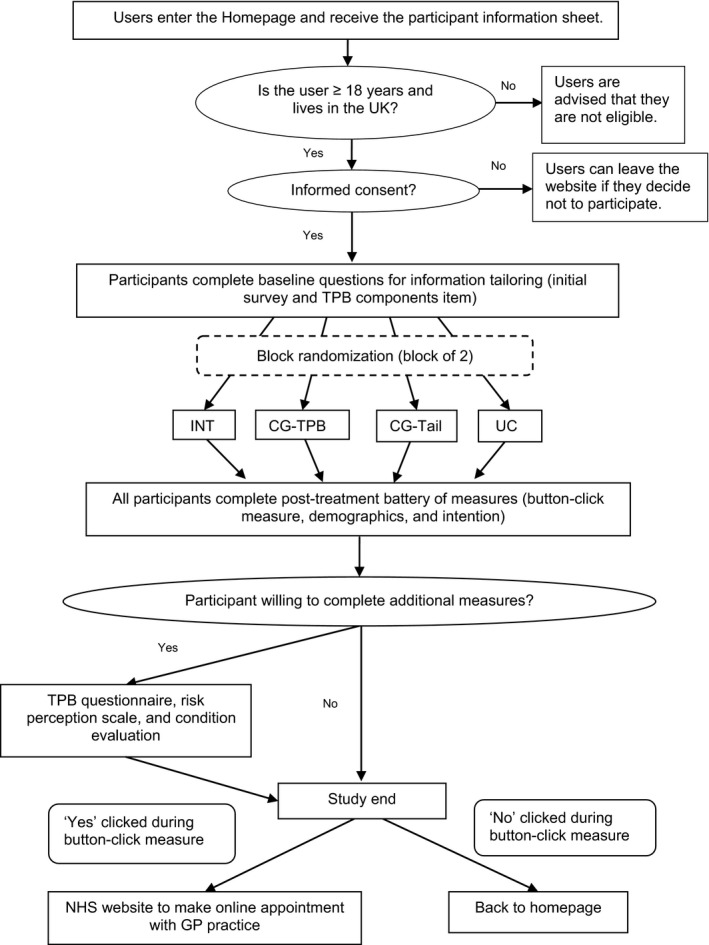
This flowchart details the study procedure. TPB = Theory of Planned Behaviour. INT = Intervention group, CG‐TPB = Control group (untailored + TPB components), CG‐TAIL = Control group (tailored + no TPB components), UC = usual care, based on Roy Castle website on lung cancer symptoms [6].

Beliefs about the outcomes of help‐seeking were targeted by showing quotes from health professionals that emphasize positive outcomes of help‐seeking (Appendix B: Figure 1), thus using the BCT ‘Information regarding behaviour, outcome’. Furthermore, the quote is endorsed by a credible source and presents a pro‐argument for early presentation (improved treatability), and thus constitutes the BCT ‘persuasive communication’.

#### Normative beliefs

**Figure 2:**
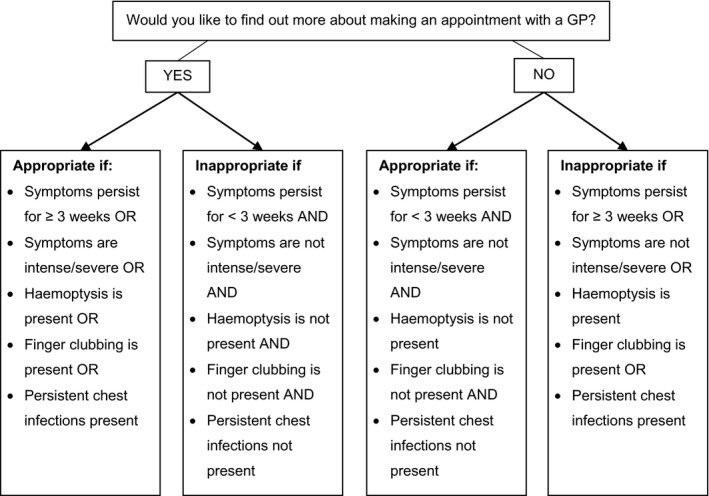
Illustration of cases in which button‐click behaviour is considered appropriate or inappropriate.

Based on the literature, we identified two groups of people whose acceptance or sanctioning of help‐seeking seems to shape individuals’ normative beliefs: significant others (e.g., family and friends) and health professionals. Users were presented with quotes from (fictional) family members and health professionals, stating that they endorse early help‐seeking (Appendix B:Figure 2). These quotes make use of the BCT ‘Social processes of encouragement, pressure, support’.

#### Control beliefs

**Figure 3:**
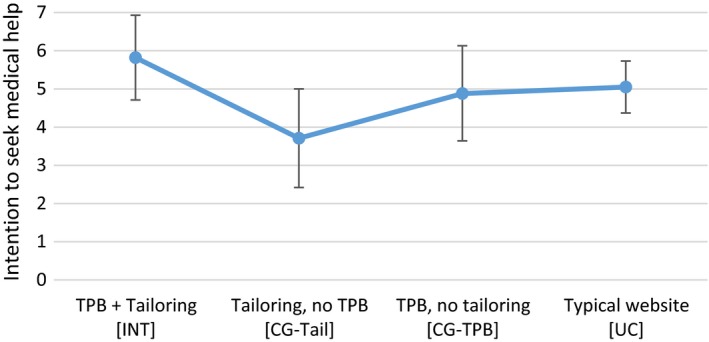
Line graph showing the mean intention to seek medical help (7‐point scale) of the four study conditions. Error bars are 95% CIs. [Colour figure can be viewed at wileyonlinelibrary.com]

To enhance perceived behavioural control over help‐seeking, a step‐by‐step guide to making an appointment was provided (Appendix B: Figure 3). This TPB component aimed to enable users to set specific goals and enhance their confidence in achieving these goals by highlighting helpful resources. Thus, this component made use of the BCT ‘Increasing skills: problem‐solving, decision‐making, goal‐setting’.

### Step 3: Tailoring

On entry to the website, users completed a set of questions designed to elicit data which were then used for information tailoring.

#### Tailoring based on symptoms and risk factors

Users were asked to report which symptoms they experienced (and whether symptoms had lasted <3 weeks or ≥3 weeks, and whether they experienced the symptom as very intense/severe), their age, and smoking status. The website then presented detailed information to participants on symptoms and risk factors they endorsed (Appendix B:Figures 4 and 5). Additionally, the intervention outputted a summary listing all risk factors and symptoms a user reported, including tailored information on whether a referral for an urgent chest X‐ray might be indicated based on NICE clinical guidelines (National Institute for Health and Care Excellence, 2015) (Appendix B:Figure 6).

#### Tailoring based on TPB constructs

On entry to the intervention website, participants were presented with the following list of statements and asked ‘Which of the following statements do you agree with most?’:

a Making an appointment to see a doctor about these symptoms would be pointless/harmful/bad.
b It is important whether others (family and friends) think it is necessary to see a doctor about these symptoms.
c It is important whether doctors think the symptoms are worth investigating.
d Getting an appointment to report these symptoms to a doctor would be difficult.

The statements represent behavioural beliefs, normative beliefs about family/friends, normative beliefs about health professionals, and control beliefs, respectively. This question aimed to identify which beliefs were most likely to impede an individual's help‐seeking behaviour, to present users with the relevant TPB component.

#### Tailoring based on user: Affected individual (AI) or proxy?

We provided information both for users who were researching their own symptoms and those searching on behalf of someone else (‘proxy’). While all users received the same information, the wording was adjusted for proxies, for example ‘If you have been coughing…’ was adjusted to ‘If your friend/relative has been coughing…’

### Step 4: Think aloud evaluation

Once a first version of the website was completed, we conducted a Think Aloud evaluation to assess whether the website was easy to navigate and whether it was acceptable and engaging. In the Think Aloud paradigm, users navigate a website while voicing their thoughts and vocalizing their actions, thus helping the researcher to identify useful and less useful/confusing features (Krug, 2013; Lewis, 1982).

#### Participants

Five users participated in this Think Aloud evaluation, aged 22–55 years (3 female, 2 male). Participants were healthy volunteers with no symptoms and were recruited from the University of Manchester and a local community group via word‐of‐mouth.

#### Procedure

Participants were presented with symptom vignettes (Box 3) and asked to imagine they (or someone in their family) were experiencing the symptoms described and to use the website to appraise the symptoms while verbalizing their thoughts.

Box 3 **Example of a symptom vignette shown to users during the Think Aloud evaluation**

1 A family member of yours is 56 years old and has been smoking for the past 20 years. She's always had a smoker's cough, but lately it has become a little worse and sounds ‘barking’. You have tried to persuade her to go see a doctor, but she refuses because she says you should not go to a GP with a mere cough. She insists it is only a smoker's cough and she doesn't want to waste the doctor's time. You first noticed these changes about a month ago.

#### Analysis

While participants completed the intervention, a researcher took notes on any comments made and on any pages/elements which caused confusion or hesitation. Following the evaluation, we assessed comments and suggestions against five criteria developed by Curtis *et al*. (2015) and implemented user suggestions if they were (1) relevant to the target behaviour, (2) available online, (3) sufficiently easy to implement, (4) aligned with usability and user experience recommendations, and (5) supported by theoretical findings and/or evidence. Suggestions were also assessed against the guiding principles (Box 2).

#### Results and conclusions

The website took 11–17 min (average 14 min) to complete, which users deemed acceptable. As Table 4 shows, feedback from the Think Aloud evaluation prompted changes to the phrasing of measures and intervention content, the structure, visual design, and information regarding credibility. Solutions proposed by the participants helped us to resolve problems in a manner acceptable to users. The evaluation also allowed us to identify what material is likely to be read in detail, what is more likely to be skimmed, and how users assess the credibility of the website.

**Table 4:** Summary of feedback received and observations made during the Think Aloud evaluation

Problems	How addressed
Acceptability and salience of information	
Would just skim over TPB quotes, though when realised it was quotes by a consultant, paid more attention	Name and role of the person belonging to the quote were highlighted to stand out
The summary is helpful because it provides details on the symptoms, their context, and the outcome (advice)	No changes required
The symptom information seems important so I would not skim this but read it properly	No changes required
Would just skim the risk factor information, as I feel I already know this	No changes required, but interesting to note
Credibility	
Was the information checked by any health professionals, or just researchers from the University? If the former, it should say that.	This information was added to the ‘About us’ page
It's not very clear that it is a UK website and based on NICE guidelines.	This was emphasized on the study homepage
Phrasing	
‘Did the [symptom] *come on suddenly or is it very severe?*’ the ‘come on suddenly’ might be difficult to answer, and difficult to distinguish from ‘severe’	This was changed just to ‘Is the [symptom] very severe?’ The point of this question was to identify people who have not had their symptom for 3 weeks but whose symptom should still be presented due to urgency. Removing the first part of the question did not change this and made it clearer/less confusing
In the question on tiredness, you should add ‘Have you felt tired *for unexplained reasons*?’	This was added, as participants may be more tired for normal reasons such as lack of sleep
There should be more options to answer ‘not sure’ on the page with questions on symptoms, for users filling the form in on someone else's behalf	Options were added where symptoms are not easily apparent to proxies, for example haemoptysis
In the question ‘Have you experienced a change in a long‐standing cough?’ the ‘long‐standing’ should be emphasised as it is otherwise easily missed	‘long‐standing’ was highlighted in bold
When asking participants whether they would like to complete the optional questionnaire at the end, the option ‘Sure, I'll help’ sounds too informal; might be off‐putting for older users	This was changed to ‘I'd like to help’, as suggested by the participant.
Website structure	
Perhaps the ‘Print summary’ option should be at the very end	This option is on the last of the information pages, thereafter only questionnaire pages follow. We were unable to add this to the very last page, as this would be the optional questionnaire
The end of the study is quite abrupt, it's unclear when it's finished	We added a message that appears at the end, telling participants that they have now completed the study and will be redirected.
The final questionnaire is too long, and the questions seem redundant	We shortened the TPB questionnaire to one item per construct
Visual design	
The quote under the image of the doctor is too close, there should be more space	The space was increased
The notification for missed question works well	No changes required
The 2^nd^ page of the final questionnaire looks the same as the first, which might be confusing	We added a banner to the top which states ‘Page 1’ and ‘Page 2’
Minor grammar/spelling/oversights	
In a few places, we had not adjusted the wording to proxies	Wording was adjusted appropriately
‘Have you experienced any expected weight loss?’ This should say *un*expected weight loss	Changed to *un*expected weight loss

### Summary of initial development

To summarize, we developed a tailored, theory‐based intervention to encourage early help‐seeking for symptoms potentially related to lung cancer, through a series of development–evaluation–development cycles using elements of the person‐based approach (Yardley *et al*., 2015). The intervention is tailored to users’ individual symptoms and risk factors and provides tailored advice on whether medical help should be sought. Furthermore, components were added to target beliefs and thereby increase intention to seek help. The final stage of the intervention development involved feasibility work which is discussed in the next section.

### Feasibility study

#### Aim

The main aims for this study were to determine

- Whether it would be possible to recruit the target population of people with undiagnosed symptoms potentially related to lung cancer,
- Whether the intervention components worked together smoothly, and
- Whether there were design issues that would compromise the validity, reliability, or objectivity of a controlled study.

## Methods

### Study design

Participants were randomized to one of four groups, comprising an intervention group receiving tailored information and TPB components (INT), a comparison group receiving untailored information with TPB components (CG‐TPB), a comparison group with tailored information but without TPB components (CG‐TAIL), and a ‘usual care’ group that received paraphrased information from an existing webpage about lung cancer symptoms (The Roy Castle Lung Cancer Foundation, n.d).

### Procedure

The study procedure is shown in Figure 1.

### Participants

We aimed to recruit people with potential lung cancer symptoms and proxies searching on their behalf, as a previous study indicated that others often research symptoms on behalf of patients with lung cancer (Mueller, Jay, Harper, & Todd, 2017).

### Eligibility

We recruited participants who were above the age of 18, lived in the United Kingdom (as information disseminated in the intervention was based on UK guidelines) and who were experiencing (or had a friend/relative with) any of the following undiagnosed symptoms:

○  A cough
○  A long‐standing cough that changes or gets worse
○  Dyspnoea
○  Discomfort in the chest, shoulder, or back
○  Haemoptysis
○  Hoarseness
○  Wheezing
○  Unexplained weight loss or unexplained loss of appetite
○  Swelling or lumps in face and/or neck area
○  Persistent/recurring chest infections
○  Fatigue
○  Finger clubbing

### Recruitment

Recruitment strategies included mailing lists (e.g., mailing lists for University staff/students, staff members of local businesses, and social clubs) social media, advertising pages (e.g., gumtree), and various websites (e.g., a website for senior citizens). Via these channels, a brief description of the study was disseminated, including the study link. The study webpage provided further details, including the participant information sheet. We also used Google Ad Words, an online advertising service which allowed us have our study website displayed near the top of Google search results when search terms related to symptoms of lung cancer were entered, such as ‘persistent cough’.

### Outcome measures

#### Primary outcome (Button‐click behaviour)

At the end of the session, participants were asked ‘Would you like to find out how to book an appointment with your doctor now?’, and we recorded whether participants clicked ‘yes’ or ‘no’ in response. Clicking ‘Yes’ would redirect participants to the NHS website for making appointments online (NHS Choices, n.d). This constitutes a behavioural proxy measure as it involves clicking a button to perform an action that is indicative of the target behaviour of making an appointment with a doctor.

Specifically, we were interested in the proportion of participants who *appropriately* click this button, as help‐seeking may not be appropriate in cases with mild or short‐term symptoms. Our categorization of when help‐seeking was considered appropriate was based on details provided by participants regarding their symptoms and risk factors (Figure 2) and was informed by guidelines developed by the UK National Institute for Health and Care Excellence for management of suspected cancer (National Institute for Health and Care Excellence, 2015), as well as discussions with a General Practitioner and consultant medical oncologists.

#### Intention

Participants were asked: ‘Do you intend to make an appointment with your doctor to have your symptoms checked?’ (Yes/No).

#### Demographics

Age, gender, ethnicity, and educational level were assessed by self‐report.

#### TPB questionnaire (optional)

The TPB questionnaire assessed intention to make an appointment with a doctor to have the symptoms checked, as well as behavioural attitude, subjective norms, and perceived behavioural control over this behaviour on a 7‐point scale (Appendix C), using the structure and wording recommended by Ajzen (Ajzen, 2006).

#### Free‐text comments box (optional)

Participants were invited to leave comments about the study or website in a free‐text box.

#### Google analytics

Data on website usage were collected via Google Analytics (Google, n.d).

### Analyses

All statistical tests were carried out using a significance level of α  =  .05 and the statistical software package IBM SPSS Statistics version 22. The proportion of appropriate button clicks was calculated for each intervention arm, and chi‐square tests for independence (or Fisher's exact test where expected frequencies were below 5) were used to test for significant differences between study groups in the proportion of appropriate clicks. The non‐parametric Kruskal–Wallis test was used to test for significant differences between the four conditions on continuous and ordinal data, and, if significant, followed up with Dunn's multiple comparison post‐hoc test (Dunn, 1964). Participants’ free‐text responses in the comments box at the end of the study were analysed using thematic analysis (Braun & Clarke, 2006).

## Results

### Sample characteristics

Between November 2015 and January 2016, 2,463 users visited the study website. Overall, 76.0% left the page without any further interactions; 24% (approx. 614 users) remained on the page and undertook further interactions, such as clicking on the ‘About’ link or clicking the ‘consent’ button.

In total, 130 participants completed the study. Participant‐reported demographics are shown in Table 5. The mean age of the sample was 49.76 years (*SD* = 15.19), ranging from 18 to 83 years. Differential dropout occurred, with more participants retained in the UC group (*n* = 54) than in the remaining three groups (INT *n* = 25, CG‐TPB *n* = 23, and CG‐TAIL *n* = 28). Out of 130, 116 participants (116/130, 89.2%) used the website for their own symptoms, and 14 (14/130, 10.8%) used it on behalf of someone else. The most commonly reported symptom was cough, followed by chest/shoulder/back pain, fatigue, dyspnoea, and wheezing (Table 5). More than a third of the sample (47/113, 36.2%) had reportedly already seen a doctor about the symptoms. Based on the criteria shown in Figure 2, help‐seeking was considered appropriate in 98.5% (128/130) of the sample.

**Table 5:** Self‐reported demographic data of the sample (*N* = 130)

	*n*	%
Sex		
Male	40	30.8
Female	90	69.2
Education level		
None	3	2.3
Primary School	0	0.0
Secondary School	43	33.1
Post‐secondary School, for example A levels	31	23.9
Undergraduate degree	28	21.5
Post‐graduate degree	25	19.2
Ethnicity		
White	121	93.1
Asian	6	4.6
Black	1	0.8
Prefer not to say	2	1.5
Smoking status		
Never smoker	51	39.2
Ex‐smoker	54	41.5
Smoker	25	19.2
Symptoms		
Cough	106	81.5
Chest/shoulder/back pain	89	68.5
Fatigue	82	63.1
Breathlessness	73	56.2
Wheezing	68	52.3
Hoarseness	49	37.7
Change in an existing cough	39	30.0
Coughing up blood	28	21.5
Recurring chest infections	19	14.6
Unintentional weight loss	18	13.8
Finger clubbing	16	12.3
Swelling in face/chest area	11	8.5

### Did the groups differ prior to receiving treatment?

Because differential dropout occurred, we tested whether study groups differed on demographic variables. A Kruskal–Wallis test showed that there was no significant difference in age, χ^2^(3) = 4.42, *p *=* *.22, and education level, χ^2^(3) = 0.72, *p *=* *.87. Using the chi‐square test, we found no significant differences between the four groups in terms of gender, χ^2^(3) = 0.49, *p *=* *.92, nor self‐reported ethnicity, χ^*2*^(9) = 4.54, *p *=* *.87 nor smoking status, χ^2^(9) = 4.54, *p *=* *.87.

### Button‐click measure

The proportion in each group who clicked the button appropriately (categorized using the criteria shown in Figure 2) in INT, CG‐TPB, CG‐TAIL, and UC, respectively, was 20% (5/25), 4.3% (1/23), 14.3% (4/28), and 11.5% (15/54). Using Fisher's exact test, group allocation was not significantly related to the proportion of appropriate clicks, χ^2^(3) =* *3.40*, p = *.37. When asked whether they intended to make an appointment with a doctor, 65.4% (85/130) clicked ‘yes’.

### Optional measures

The Kruskal–Wallis test showed no significant differences between the four groups in behavioural attitude, subjective norm, or perceived behavioural control (*p *>* *.05). The four conditions differed significantly in scores on the intention variable, χ^2^(3) = 8.14, *p *=* *.04, with the highest intention reported among participants in the INT group (Figure 3). Dunn's multiple comparison test showed a significant difference between INT and CG‐TAIL (*p *=* *.03).

### Open‐ended comments

Thirty‐one participants wrote a comment following study participation.

#### (i) Positive feedback

Fourteen people commented positively.

Some users reported that using the website had changed their beliefs regarding help‐seeking.

Thank you for developing this tool; I have had a ‘nagging doubt’ that something might be wrong (hoping it's not lung cancer) for a while, but did not want to waste my doctor's time as I didn't think my symptoms were ‘strong enough’. Its [sic] good to get an ‘independent’ recommendation on whether an appointment should be made or not.

[INT]

Some also suggested that using the website had encouraged them to mention symptoms they had not thought relevant before.

I was given antibiotics so feel reassured at the moment but I have noticed and complained about the changes in my fingers and nails for about 2 weeks but didn't mention this to the GP as I didn't think it relevant. If my cough persists after I have finished the antibiotics I will go and tell the GP about the changes to my fingers.

[INT]

#### (ii) Suggestions for improvement

Ten people suggested room for improvement.

Some suggested that the health information provided could make people anxious.

I think it's a good idea to have a tool like this as it may encourage people with long standing symptoms like these to go and see their GP but caution should be used as you run the risk of making people unnecessarily anxious about their symptoms or condition.

[UC]

Several users criticized that the website did not take pre‐existing conditions into account.

I was diagnosed with bronchiectasis [i]n 2007. Referring back 3 weeks in my life therefore does not give a true picture for the purpose of the tool which seems to be directed to people with cough and no previous diagnosis. Some of the questions therefore give an inaccurate flow and response.

[INT]

Finally, some users said the website had not answered their questions.

My chest feels very heavy and painful quite frightening but even though my father brother and aunties and uncle all died of heart attacks when I was checked I was told I was fine but if it continues for a few hours might phone as my back is hurting too. This site hasnt [sic] answered any of my question [sic] of when I should phone for help.

[UC]

#### (iii) Previous experiences with health services

Several people (*n* = 10) mentioned that they had already seen a GP; five of these were reportedly not satisfied with the advice received so far.

My GP is useless. When I visited him with my symptoms he was not ready to carry out further tests. I felt threatened by his rude attitude. I will never see him again even my symptoms gets [sic] worse.

[CG‐TPB]

One person reported that his friend had previously received less medical attention from his General Practitioner for his symptoms because of his smoking habit.

The idea is good one problem is my friend who smokes will not make an appointment as the GP bangs on about smoking, how he should pack up, what he should be doing and refers him to the ceasation [sic] nurse. The symptons [sic] of what he went with initially are overlooked while he feels like he is being preached at about his smoking habit.

[CG‐TPB]

Difficulties obtaining an appointment due to limited resources were also reported.

Tried to get urgent appointment yesterday. Fully booked for weeks. Told to go A&E!

[UC]

#### (iv) Button‐click measure

Comments indicate some people did not use the button‐click as they did not feel the need for further assistance with making an appointment.

I did not ask you to help me make an appointment with my doctor as it is very easy for me to do this, all I have to do is lift the telephone. Thank you for your help.

[CG‐TAIL]

## Discussion

We present in this study a detailed account of the steps we undertook to develop a user‐centred, evidence‐based intervention, highlighting how we combined qualitative research with psychological theory. This detailed description can be used as a method for supporting similar intervention design. This is important because information on the content and design of interventions for health behaviours is often cursory, superficial, or lacking entirely (Glasziou *et al*., 2008; McCleary *et al*., 2013). This makes findings difficult to reproduce or implement in practice and hinders other researchers from building on previous work (Wood *et al*., 2015).

A key strength of our intervention development is that we incorporated elements of the person‐based approach (Yardley *et al*., 2015) by drawing on our interview study with recently diagnosed patients with lung cancer who used the web to research their symptoms, other previous studies with lung cancer patients, Patient and Public Involvement work, a Think Aloud evaluation, and using guiding principles to ensure the intervention remained focused on its key objectives. Further strengths of the intervention development process were the combination of the person‐based approach with theory, and the use of the Behaviour Change taxonomy (Michie *et al*., 2008) to identify specific, observable mechanisms to bring about changes in behaviour.

The feasibility study assessed whether it would be possible to recruit the target population of people with undiagnosed symptoms potentially related to lung cancer and whether the intervention components worked together smoothly.

Of those who showed interest in our website (by engaging in some form of interaction after viewing the homepage), 21.2% completed the study. Low participation levels of between 10 and 12% have been noted in previous web‐based interventions (Paul *et al*., 2017; Peels *et al*., 2012), and some even report levels as low as 0.24% (Koo & Skinner, 2005), although others report more positive experiences (Murray *et al*., 2009). Additionally, online trials have often been associated with low retention levels (Mathieu, McGeechan, Barratt, & Herbert, 2013; Murray *et al*., 2009). These low participation and retention levels highlight that it is important to publish details regarding online intervention design; more systematic, informed approaches are needed to ensure interventions are feasible and acceptable to users. A meta‐analysis of tailored, web‐based health interventions indicates, however, that high attrition rates do not necessarily bias study outcomes (Lustria *et al*., 2013).

Overall, the study was successful in recruiting the target population. Over a 3‐month period, we recruited 130 participants who reported symptoms related to lung cancer, and in the vast majority (98.5%), symptoms were either prolonged (≥3 weeks), severe, or key warning signs (e.g., haemoptysis). This demonstrates the feasibility of recruiting participants with relevant undiagnosed symptoms, thus evaluating a help‐seeking intervention in an ecologically valid setting. However, it should be noted that 76% of website visitors left the website without further interaction. A high bounce rate may indicate low user satisfaction (Sculley, Malkin, Basu, & Bayardo, 2009). Because browsers do not allow forced redirection on exit of a website, it was not possible to elicit further details from users who left the page. Possibly, users clicking on our Ad Word link expected a service rather than study participation. It is perhaps unsurprising that users were unwilling to participate in a study, even if it involved only a brief, single‐session intervention, as those researching symptoms online may be feeling anxious (White & Horvitz, 2009).

It should be noted that our sample differed on several variables from what would be expected from a lung cancer population. Our sample had an average age of 49.8 years, whereas lung cancer is most prevalent among those aged 71–80 years (British Lung Foundation, 2016). Compared to the UK lung cancer population, our sample also included a higher self‐reported proportion of non‐smokers (40%, vs. 20%, Cufari *et al*., 2017) and females (69%, vs. 46.2%; Office for National Statistics, 2016). This raises concerns about generalizability to the lung cancer population and highlights the difficulties in reaching the target population. These differences may also be present because we included those researching symptoms on behalf of others. Although we incorporated strategies to engage those at high risk (e.g., by targeting older people and smokers during recruitment and ensuring the website met criteria for senior‐friendliness), these groups remained difficult to engage. Future endeavours to harness the web in encouraging earlier presentation should focus on strategies to target at‐risk groups. It should also be noted that some barriers to web use among older people will inevitably decrease in future (Mueller, Jay, Harper, & Todd, 2017; Zickuhr & Madden, 2012).

We recruited only a small number of Black and Minority Ethnic (BME) users. The difficulty of recruiting and engaging BME participants in cancer studies is well documented in the literature (Lai *et al*., 2006; Pinsky *et al*., 2008).

### Lessons learned

#### Differential dropout in online interventions

The main design issue identified in the feasibility study pertains to the differential dropout of participants across the four study conditions. Differential dropout can limit the validity of findings (Moher *et al*., 2010), particularly when attrition occurs for systematic rather than random reasons (Bell, Kenward, Fairclough, & Horton, 2013).

Possibly, differential dropout occurred due to differing lengths of the four conditions. The UC group retained approximately twice as many participants as the remaining three groups, and this group also received the lowest amount of information and fewest website pages. Research has shown that Internet users prefer brief, concise information for web‐based health interventions (Yardley, Morrison, Andreou, Joseph, & Little, 2010).

Thus, to mitigate the risk of differential attrition, online trials should ensure all study groups receive approximately the same number and length of information pages, and this should be kept as concise as possible. This is particularly relevant for tailored interventions which are increasingly popular in online health behaviour change strategies (Lustria *et al*., 2013), because tailored and untailored groups may differ in length or amount of information. Furthermore, future studies should involve thorough analyses of the acceptability of different study groups for different subgroups prior to implementing an intervention in an evaluation study, in order to premeditate and mitigate differential dropout.

#### Measuring help‐seeking behaviour

We attempted to measure help‐seeking behaviour using a behavioural proxy, that is, whether participants clicked a button to make an appointment online or not. Only 13 people (0.1%) across all four conditions clicked ‘Yes’ on the ‘button‐click’ measure, although 65.4% indicated they intended to make an appointment with a doctor. This indicates that the button‐click measure did not succeed as a measure to assess help‐seeking behaviour.

Novel geo‐tracking techniques might prove useful in the future to assess whether Internet users access health services following online searches (White & Horvitz, 2013); however, the algorithms used to detect health care utilization require further refinement and validation before they can provide useful insights (Mueller, Jay, Harper, Davies, *et al*., 2017). Until such validated measures are available, researchers may need to resort to measuring behavioural intention. Many studies have provided evidence for the value of predicting behaviour using intentions, although results vary depending on the behaviour under study (Ajzen, Albarracin, & Hornik, 2007; Ajzen & Fishbein, 1980; Albarracín, Johnson, Fishbein, & Muellerleile, 2001; Godin & Kok, 1996; Hausenblas, Carron, & Mack, 1997; Sheeran & Orbell, 1998).

#### Supporting people who have already sought help

Participants’ comments showed that some users of the website had already sought medical advice and were dissatisfied with the care they received. Previous research shows that people with lung cancer often present to health services multiple times prior to diagnosis (Lyratzopoulos, Abel, McPhail, Neal, & Rubin, 2013; Lyratzopoulos, Neal, Barbiere, Rubin, & Abel, 2012). Those searching for information on lung cancer symptoms may benefit from information on when symptoms require further investigation and when to re‐present following initial consultation.

#### Preliminary findings

Findings from our feasibility study should be interpreted with caution, as this study was designed to assess feasibility rather than testing for effects of the intervention, and therefore, the relatively small sample (*N* = 130) was not powered for inferential statistics. We found a significant difference in intention to make an appointment between the intervention group which received tailored, TPB‐based information and the control group which received tailored but non‐theory‐based information. This finding is interesting because these two groups were identical excepting only the TPB components. Thus, this finding suggests a causal role of TPB components in intention to seek medical advice, but this needs to be confirmed in a fully powered trial before drawing firm conclusions. It should also be noted that changes in intention do not necessarily translate into changes in behaviour. While intention has been shown to predict some behaviours with high accuracy (Armitage & Conner, 2001), intention is not always an accurate predictor of actual behaviour (Rhodes & Dickau, 2012; Rhodes & Plotnikoff, 2006; Webb & Sheeran, 2006). It is also interesting to note that, while we found changes in intention, we did not find changes in the variables that determine intention according to the TPB. It is possible that our measurement of these determinants was not suitable or that other factors play a role, such as knowledge (de Nooijer, Lechner, & de Vries, 2003).

### Conclusions

We present a detailed description of our intervention development process, thus providing useful insights for future researchers intending to develop evidence‐based, theory‐based, user‐centred online health interventions. Moreover, we can conclude from the feasibility study that it is possible to recruit participants with undiagnosed symptoms for a help‐seeking intervention using online recruitment strategies. This information will be of use to future help‐seeking and early detection trials. It will also be relevant to studies examining web use for symptom appraisal; a previous systematic review shows that experimental studies tend to use fictional symptom scenarios (Mueller, Jay, Harper, Davies, *et al*., 2017), but this study shows that it is possible to investigate web use prior to diagnosis in a more ecologically valid setting. Finally, the feasibility study highlights that the intervention needs to be adapted to provide more support for people who have reportedly already sought help, but felt dismissed by health care professionals. The feasibility study also provides some preliminary evidence that our ‘TPB components’, which were designed to target beliefs about help‐seeking, were effective in increasing intention to seek help for symptoms, but this finding should be interpreted with caution as the study was not powered for inferential statistics. Further validation in a fully powered randomized trial is necessary and currently underway.

## Conflict of interest

All authors declare no conflict of interest.

## Figures and Tables

**Figure B1:**
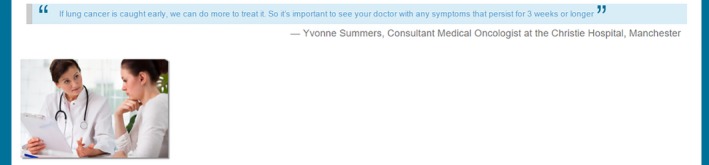
Screenshot showing how we targeted behavioural beliefs on the intervention website. [Colour figure can be viewed at wileyonlinelibrary.com]

**Figure B2:**
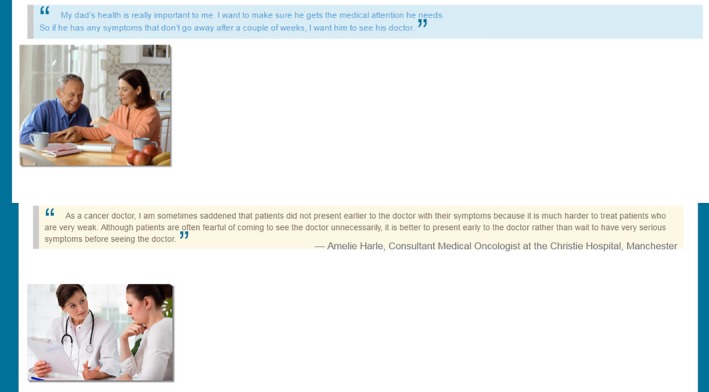
Screenshot showing how we targeted normative beliefs about family/friends (top) and health professionals (bottom) on the intervention website. [Colour figure can be viewed at wileyonlinelibrary.com]

**Figure B3:**
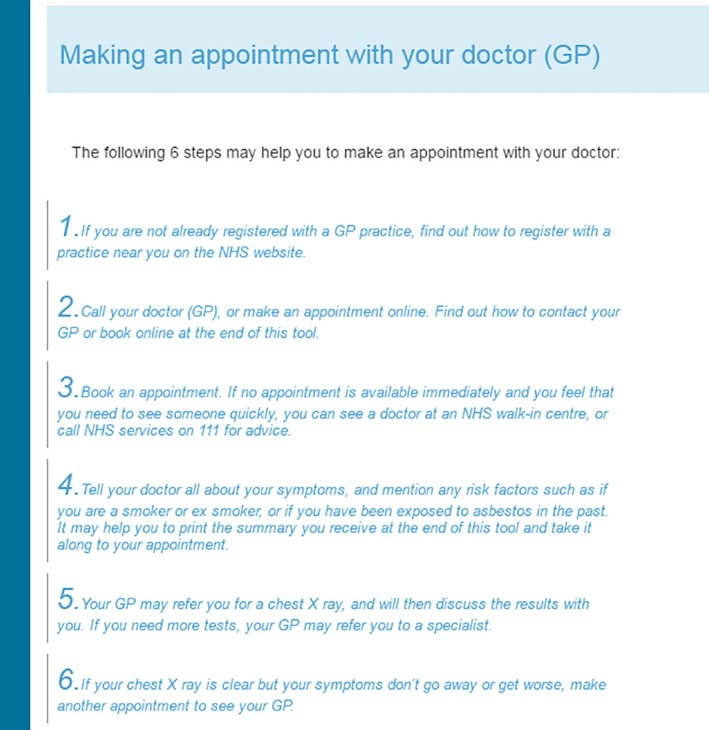
Screenshot showing how we targeted control beliefs on the intervention website. [Colour figure can be viewed at wileyonlinelibrary.com]

**Figure B4:**
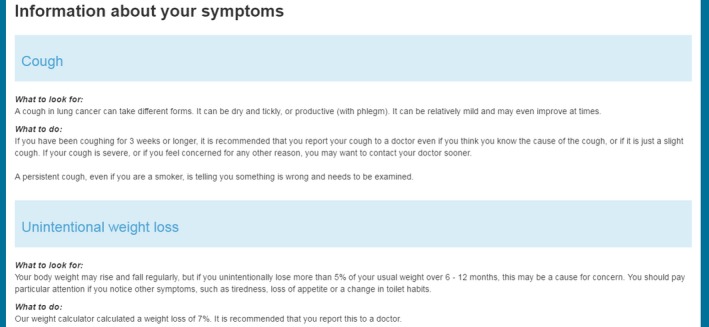
Screenshot shows an example of tailored symptom information a user would receive who endorsed a cough and unintended weight loss during the initial survey. [Colour figure can be viewed at wileyonlinelibrary.com]

**Figure B5:**
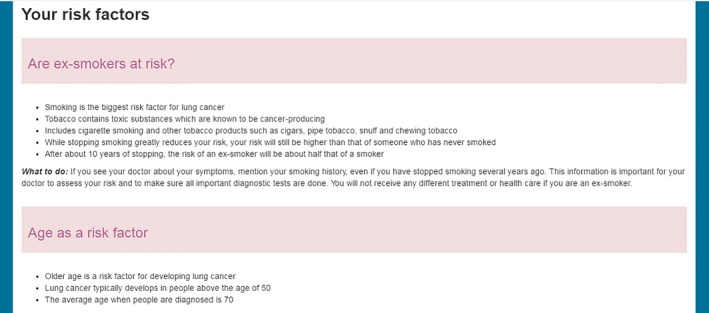
Screenshot shows an example of tailored risk factor information a user would receive who reported being an ex‐smoker and aged above 40. [Colour figure can be viewed at wileyonlinelibrary.com]

**Figure B6:**
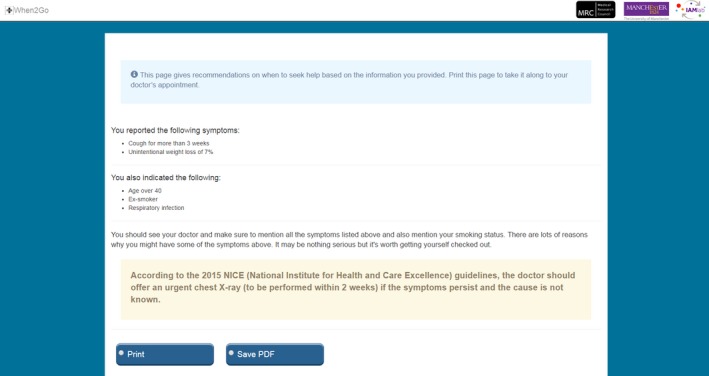
Screenshot of the tailored intervention summary. [Colour figure can be viewed at wileyonlinelibrary.com]

## Appendix A Construct domains and relevant behaviour change techniques identified from Michie *et al*. (2008)

### A.1

**Table:**

Construct domain	Maps on to TPB construct	Behaviour change techniques	Suitability
Beliefs about consequences	Behavioural beliefs	Self‐monitoring	Not suitable This would have to involve monitoring over a longer period of time – not amenable to mode of delivery
		Information regarding behaviour and outcome;	Suitable
		Persuasive communication	Suitable
		Feedback	Not suitable This would have to involve monitoring over a longer period of time – not amenable to mode of delivery
Social influences	Normative beliefs	Social processes of encouragement, pressure, and support	Suitable
		Modelling/demonstration of behaviour by others	Not suitable Not suitable for the targeted behaviour; difficult to ‘show’ people making an appointment on a website; and probably not necessary as the physical act of making an appointment does not pose difficulties
Beliefs about capabilities	Control beliefs	Increasing skills: problem‐solving, decision‐making, and goal‐setting	Suitable
		Self‐monitoring	Not suitable This would involve monitoring over a longer period of time – not amenable to mode of delivery
		Graded task, starting with easy task	Not suitable This would involve monitoring over a longer period of time – not amenable to mode of delivery. Also not suitable for the targeted behaviour, as it is difficult to design ‘graded tasks’ leading up to making an appointment
		Coping skills	Not suitable Not suitable for the targeted behaviour, which only involves a phone call to make an appointment
		Rehearsal of relevant skills	Not suitable This would have to involve monitoring over a longer period of time – not amenable to mode of delivery. Also not suitable for the targeted behaviour, which only involves a phone call – rehearsal of skills not needed
		Social processes of encouragement, pressure, and support	Suitable but in this context not suitable to target control beliefs; more suitable to target normative beliefs. Based on findings from the literature, people do not lack the belief that they are *able* to complete the behaviour (making an appointment); rather they lack the belief that they can fit this in with existing responsibilities or appointment availability at their clinic (Birt *et al*., 2014). Social encouragement is unlikely to target these beliefs
		Feedback	Not suitable This would have to involve monitoring over a longer period of time – not amenable to mode of delivery

## Appendix C Design and structure of the TPB questionnaire

### C.1

**Table:**

TPB construct	Item
Behavioural intention	‘If my symptoms persisted for 3 weeks or longer, I would intend to make an appointment with my doctor to have my symptoms checked.’ On a 7‐point scale from ‘strongly disagree’ to ‘strongly agree’
Attitudes towards the behaviour	‘For me to make an appointment with my doctor to have these symptoms checked would be…’ 1 ‘pointless’ to 7 ‘useful’
Subjective norms	‘Most people who are important to me want me to make an appointment with my doctor to have these symptoms checked’ from 1 ‘strongly disagree’ to 7 ‘strongly agree’
Perceived behavioural control	‘For me to make an appointment with my doctor to have these symptoms checked would be…’ 1: ‘difficult’ to 7: ‘easy’
